# Respiratory Delivery of Highly Conserved Antiviral siRNAs Suppress SARS-CoV-2 Infection

**DOI:** 10.3390/ijms262311675

**Published:** 2025-12-02

**Authors:** Yuan Zhang, Matt D. Johansen, Scott Ledger, Stuart Turville, Pall Thordarson, Philip M. Hansbro, Anthony D. Kelleher, Chantelle L. Ahlenstiel

**Affiliations:** 1 Kirby Institute, University of New South Wales, Sydney, NSW 2052, Australia; yuanzhang@kirby.unsw.edu.au (Y.Z.); sledger@kirby.unsw.edu.au (S.L.); sturville@kirby.unsw.edu.au (S.T.); akelleher@kirby.unsw.edu.au (A.D.K.); 2 Centre for Inflammation, School of Life Sciences, Faculty of Science, Centenary Institute and University of Technology Sydney, Sydney, NSW 2050, Australia; matt.johansen@uts.edu.au (M.D.J.); philip.hansbro@uts.edu.au (P.M.H.); 3 RNA Institute, University of New South Wales, Sydney, NSW 2052, Australia; p.thordarson@unsw.edu.au

**Keywords:** SARS-CoV-2, RNA interference, siRNA, respiratory delivery, innate immune activation, immunomodulation, chemical modification

## Abstract

COVID-19 has resulted in over 777 million confirmed cases and more than 7 million deaths globally. While vaccination offers protection for individuals with a functional immune system, immunocompromised populations will not generate sufficient responses, highlighting the critical need for new antiviral treatments. Here we evaluated four highly conserved anti-COVID siRNAs targeting the ORF1a-Nsp1, Membrane, and Nucleocapsid regions by identifying their antiviral efficacy in vitro and investigated the direct delivery of naked siRNAs to the respiratory tract of mice via intranasal instillation to provide proof-of-concept evidence of their in vivo antiviral activity. Dose-response analysis of siRNAs revealed a range of IC50 0.02 nM to 0.9 nM. Intranasal administration of naked anti-COVID siRNA-18 in a K18-hACE2 transgenic SARS-CoV-2 mouse model was capable of reducing viral mRNA levels and disease severity. While anti-COVID siRNA-30 induced modest interferon-stimulated gene expression in vitro and immune cell infiltration in vivo, these effects were markedly reduced by 2′-O-methyl-AS456 chemical modification, which preserved antiviral efficacy against SARS-CoV-2 while minimizing off-target immune activation. These results demonstrate the feasibility of direct respiratory siRNA administration for in vivo viral suppression and highlight the benefit of using conserved target sequences and chemical modification to enhance therapeutic safety and efficacy.

## 1. Introduction

COVID-19 infections and mortality continue to increase globally despite the introduction of effective vaccines [1]. According to the World Health Organization, over 777 million confirmed cases of coronavirus disease 2019 (COVID-19) and more than 7 million deaths have been reported globally (https://covid19.who.int/ (accessed on 30 September 2025)). While the vaccination strategy is highly successful, certain populations may be associated with lower antibody titres and more severe clinical outcomes, including those with impaired immunity (such as immunodeficiencies [2] or autoimmune diseases [3,4]), hematological malignancies, or solid-organ transplant recipients receiving immunosuppressive therapy, as well as individuals with other predisposing factors for severe disease, such as advanced age (>75 years) [5] or chronic conditions (e.g., COPD [6], diabetes [7], hypertension [8], or smoking history [9]). In these populations, reduced antibody titers lead to a higher risk of breakthrough infection and severe clinical outcomes [10,11]. Oral antivirals have expanded treatment options; however, their clinical utility in immunocompromised patients is limited by adverse effects and clinically significant drug–drug interactions. Nearly 30% of the 50 most commonly prescribed drugs have the potential to interact with Paxlovid [12]. Hence, for these high-risk populations, there is a critical need to develop direct-acting antiviral treatments that do not rely on an intact adaptive immune response to reduce mortality and will alleviate the burden on healthcare systems via hospital admissions, specifically those that require intensive care. Further, the ongoing emergence of Variants of Concern (VOC) compromises vaccine efficacy, highlighting the necessity of mutation-resilient therapeutics [13]. The propensity of SARS-CoV-2 to adapt to environmental pressures emphasizes the necessity of developing therapeutics that are effective for both existing and emerging variants and would ideally provide pandemic preparedness for future SARS-coronaviruses.

RNA interference (RNAi), first discovered by Fire et al. in *Caenorhabditis elegans*, is a conserved biological process induced by non-coding RNA (ncRNA) that inhibits gene expression by blocking the transcription or translation of specific genes [14]. Among many small RNAs that can produce RNAi, highly conserved short interfering (si)RNA have emerged as direct-acting antiviral agents that can be rationally designed against the continuous mutation of viruses [15,16]. Our previous study identified four lead anti-COVID siRNAs as effective targets against SARS-CoV-2 infection [17].

In this study, the in vivo potential of two lead siRNAs via intranasal delivery was evaluated in the K18-hACE2 transgenic mouse model, which mimics the pathophysiological features of SARS-CoV-2 infection in humans. Respiratory administration ensures direct delivery to the primary site of viral replication while bypassing gastrointestinal metabolism, thereby reducing systemic exposure and lowering the risk of drug–drug interactions compared with oral agents. Although their efficacy is independent of host immune status, siRNAs have potential to be recognized as foreign and trigger inflammatory responses, which is undesirable, particularly in the respiratory tract. Thus, site-specific chemical modifications were explored to mitigate innate immune activation, providing an initial foundation for their future incorporation into nanoparticle delivery systems. This study aims to provide proof-of-concept support for a mutation-resilient, direct-acting siRNA strategy against SARS-CoV-2, with the potential to benefit high-risk vulnerable groups and immunocompromised populations and to strengthen preparedness for future coronaviruses.

## 2. Results

### 2.1. Conservation Analysis of Lead Candidate Anti-COVID siRNAs in SARS-CoV-1 and SARS-CoV-2 Human and Animal Sequences

**Figure 1:**
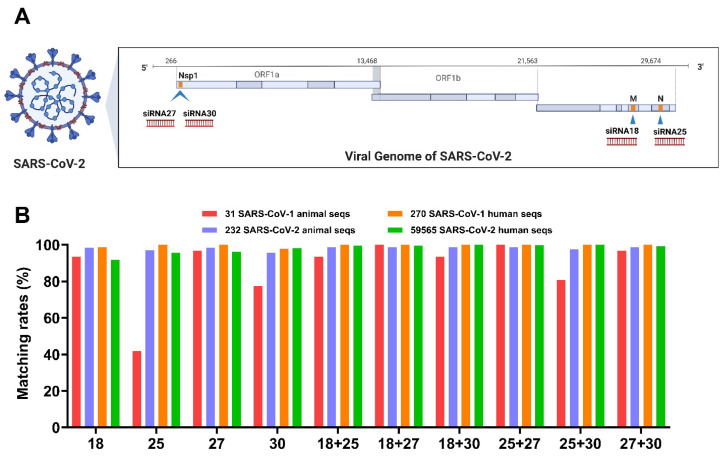
SARS-CoV-2 genome map and conservation analysis of top-four COVID siRNAs in SARS-CoV-1 and SARS-CoV-2 sequences. (**A**) SARS-CoV-2 genome and lead candidate anti-COVID siRNA targets. Locations of the four lead siRNA candidates (siRNA-18, siRNA-25, siRNA-27, and siRNA-30) were indicated. (**B**) Conservation analysis of lead candidate anti-COVID siRNAs in SARS-CoV-1 and SARS-CoV-2 updated sequences. A total of 59,565 SARS-CoV-2 seqs from 16 May 2022 to 21 April 2025 were retrieved for analysis. Also, 31 SARS-CoV-1 animal seqs, 232 SARS-CoV-2 animal seqs, and 288 SARS-CoV-1 human sequence were included.

We assessed the four lead candidates and dual combinations for sequence conservation in both human and animal sequences of SARS-CoV-1 (301 seqs, 270 human and 31 animal seqs) and SARS-CoV-2 (3242 seqs, 3010 human and 232 animal seqs). The four lead candidate siRNAs were located in the Membrane (Mem) region (27,852–27,870 bp for siRNA 18), in the Nucleocapsid (Nuc) region (30,494–30,512 bp for siRNA25), and in the Nsp1 region (378–396 bp for siRNA27 and 631–649 bp for siRNA30) (Figure 1A). All four lead candidates demonstrated a significant protective effect against SARS-CoV-2 infection in vitro [7].

The conservation of siRNA 18 across SARS-CoV-1/2 human and animal sequences was ≥91.65%, and the conservation of siRNA 27 was ≥96.12% in each group (Figure 1B). For siRNA25 and siRNA30, both showed high conservation (≥95.55%) across SARS-CoV-2 animal sequences, SARS-CoV-1 human sequences, and updated SARS-CoV-2 human sequences. In contrast, within the SARS-CoV-1 animal sequence group, siRNA30 maintained a relatively high sequence coverage of 77.42%, while siRNA 25 showed reduced coverage at 41.94%. These findings suggest that all four lead candidate anti-COVID siRNAs are highly conserved across SARS-CoV-2, and among them, siRNA 18, siRNA 27, and siRNA 30 also demonstrate strong conservation across SARS-CoV-1 related sequences. When the four candidate siRNAs were combined in pairs, each pair provided sequence coverage >80% across the SARS-CoV-1 animal sequence group and >97% across the SARS-CoV-2 animal sequence group, which means that at least one siRNA in each pair matched the viral genome in the respective sequence. Notably, in both groups of SARS-CoV-1 and SARS-CoV-2 human sequences, each dual combination of our four candidate siRNAs showed matching rates of >99%, suggesting that a combination approach provides superior sequence coverage and may have potential for providing pandemic preparedness (Figure 1B).

### 2.2. Gene Silencing Effect of Lead Candidate Anti-COVID siRNAs and Protection Against SARS-CoV-2 Infection

To enable high-throughput screening in a BSL2/PC2 laboratory setting, a total of four 293T reporter cell lines expressing the SARS-CoV-2 target regions (Mem, Nuc, or Nsp1) fused to GFP were generated, sequence confirmed, and activity verified using their specific siRNAs at 5 nM. All siRNAs—18, 25, 27, and 30—showed significant suppression of GFP expression in the 293T GFP reporter cells. In 293T GFP reporter cells, the GFP suppression induced by siRNA18 (57.90% silencing, *p* < 0.0001) and siRNA30 (61.41% silencing, *p* < 0.0001) was greater than that induced by siRNA25 (48.38% silencing, *p* < 0.0001) and siRNA27 (21.84% silencing, *p* < 0.01) (Figure S1). This shows that siRNAs induce varied gene silencing effects in different target constructs. Further, GFP suppression also appears to vary in fully matched siRNA sequences, as seen in both siRNA27 and siRNA30, which, despite being fully matched to the target virus sequence, displayed markedly different silencing efficiency.

**Figure 2:**
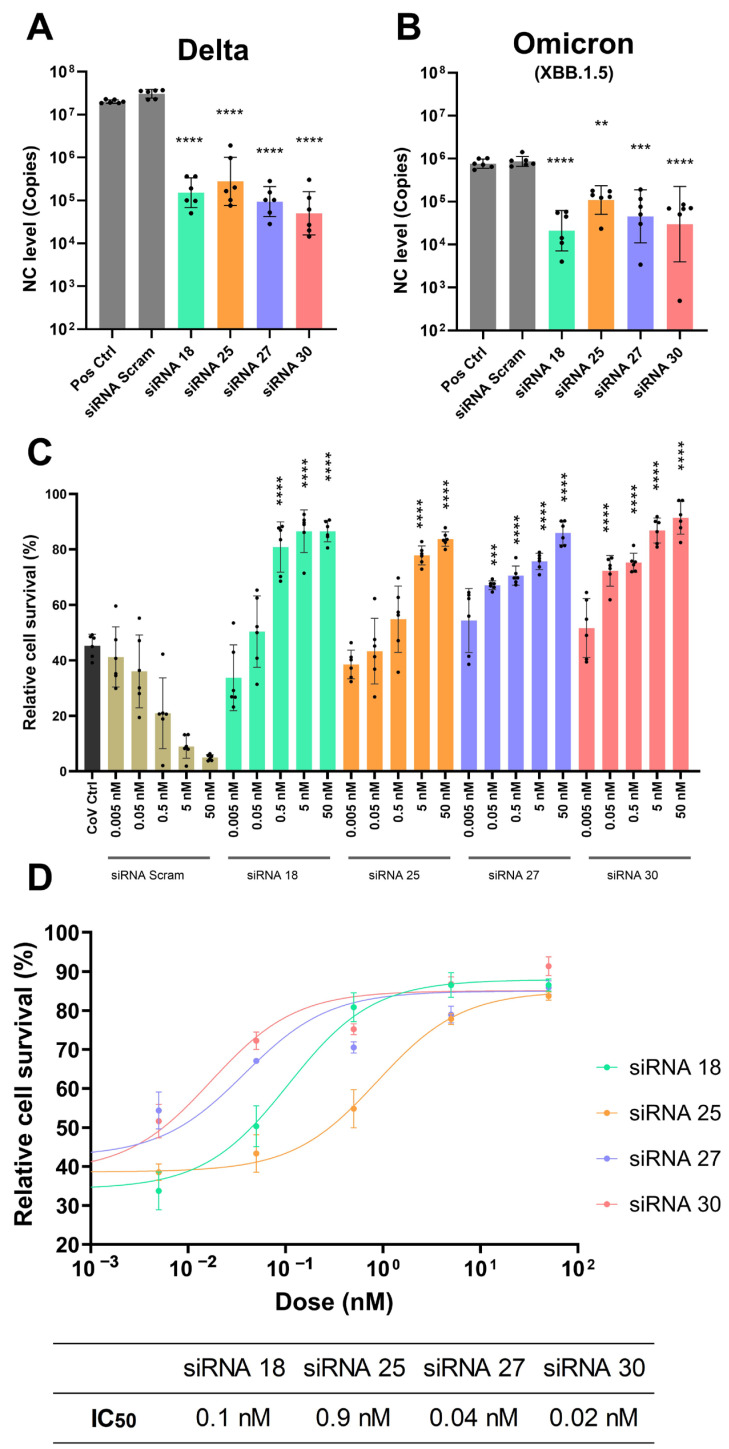
Protection effect and dose-response assay of lead candidate siRNAs against SARS-CoV-2. Protection effect of 5 nM siRNA 18, siRNA 25, siRNA 27, and siRNA 30 against SARS-CoV-2 Delta infection (**A**) and Omicron XBB.1.5 infection (**B**) in Vero E6 cells. Transfection was performed 6 h post-seeding, and SARS-CoV-2 infection with the MOI-0.05 was performed 1 h after transfection. Supernatant was collected after 3 days of treatment. (**C**) Dose-response assay of siRNA 18, siRNA 25 siRNA 27 and siRNA 30 from 0.005 nM to 50 nM in Vero E6 infected with SARS-CoV-2 Omicron XBB.1.5. Lipofectamine transfection and SARS-CoV-2 XBB.1.5 infection was performed as in (**A**,**B**). Live cells were then stained by NucBlue™ Live Cell Stain (Thermo Fisher Scientific, Waltham, MA, USA) and imaged using the IN Cell Analyzer 2500HS (Cytiva, Marlborough, MA, USA) after 3 days of treatment. (**D**) Dose-response curves of lead siRNA candidates against SARS-CoV-2 XBB.1.5 infection. Vero E6 cells were treated with serial dilutions of siRNA 18, 25, 27, or 30 (0.005–50 nM) and infected with SARS-CoV-2 XBB.1.5. The IC_50_ values were calculated from nonlinear regression curves, reflecting the concentration at which each siRNA achieved 50% of its maximal protective effect. Statistical comparisons were performed with the siRNA Scramble (Scram) control group with the same siRNA concentration. ** = *p* < 0.01, *** = *p* < 0.001, **** = *p* < 0.0001.

We then confirmed the effect of the lead candidate anti-COVID siRNAs in Vero E6 cells infected with SARS-CoV-2 Delta and Omicron XBB.1.5 variants by measuring viral load, as indicated by nucleocapsid (NC) mRNA levels. Specifically, siRNA 18, siRNA 25, and siRNA 30 decreased viral copies in the Delta variant from 7.3 to 5.2, 5.4, and 4.7 log_10_ copies, respectively, and in the XBB.1.5 variant from 5.9 to 4.3, 5.0, and 4.5 log_10_ copies, respectively (Figure 2A,B), which corresponded to their gene silencing effects in reporter cell lines.

Interestingly, although siRNA 27 exhibited limited GFP suppression in the reporter cells, achieving only 21.84% silencing of GFP expression (Figure S1), it demonstrated a potent protective effect in Vero E6 cells against the SARS-CoV-2 Delta and XBB.1.5 variants, with 1- to 2-log_10_ reduction in SARS-CoV-2 NC mRNA levels, decreasing from 7.3 to 5.0 log_10_ copies for the Delta variant and from 5.9 to 4.6 log_10_ copies for the XBB.1.5 variant (Figure 2A).

To investigate whether the potency of the lead candidate siRNAs was contributing to this varied effect, we performed dose-response assays with serial dilutions of siRNA starting at 50 nM down to 0.005 nM. The protective effect against SARS-CoV-2 XBB.1.5 infection in Vero E6 cells, as measured using a cell survival assay, was dose-dependent and increased with increasing siRNA concentration. The minimum effective dose (MED), defined as the lowest siRNA concentration at which a statistically significant reduction in viral RNA (*p* < 0.05) was observed compared to negative controls, was 0.05 nM for siRNA 27 (*p* < 0.0001) and siRNA 30 (*p* < 0.0001), 0.5 nM for siRNA 18 (*p* < 0.0001), and 5 nM for siRNA 25 (*p* < 0.0001) (Figure 2C). Corresponding IC_50_ (half maximal inhibitory concentration) values were determined to be 0.1 nM for siRNA 18, 0.9 nM for siRNA 25, 0.04 nM for siRNA 27, and 0.02 nM for siRNA 30, further confirming their relative antiviral potency (Figure 2D). As siRNAs 27 and 30, closely followed by 18, demonstrated the most potent silencing effects at the lowest doses (0.05 and 0.5 nM, respectively), we proceeded to character the protective effect of siRNA 30 and 18 in vivo.

### 2.3. The Protective Effect of siRNAs Against SARS-CoV-2 In Vivo

**Figure 3:**
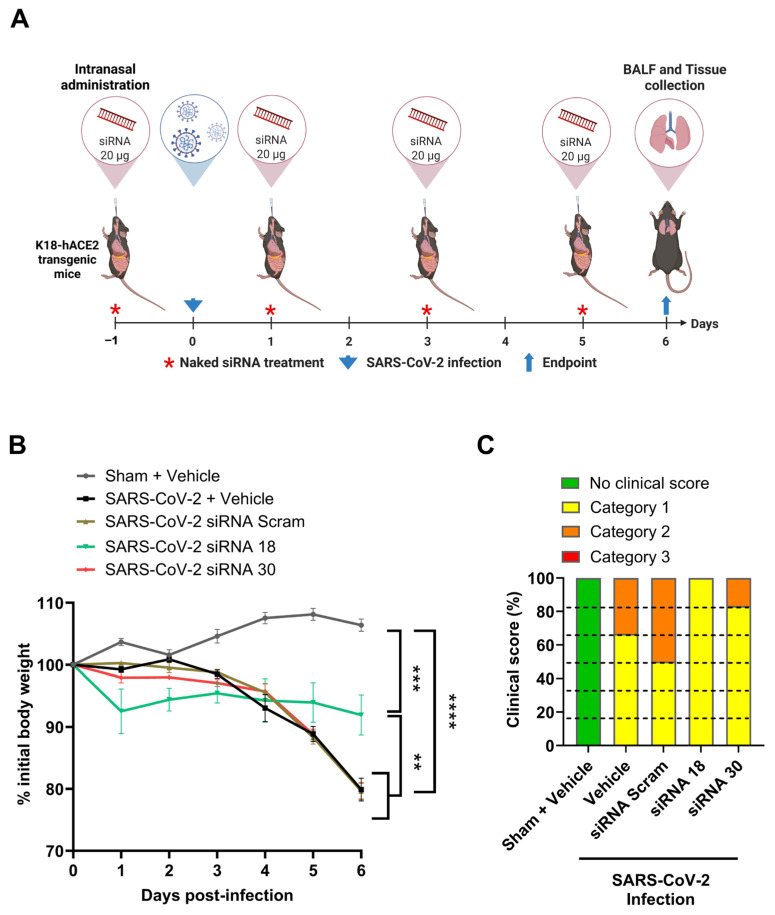
Effects of naked siRNAs on body weight and clinical scores in SARS-CoV-2–infected K18-hACE2 mice. (**A**) Schematic of siRNA intranasal delivery in K18-hACE2 transgenic mice post SARS-CoV-2 Delta infection. Five groups of K18-hACE2 transgenic mice with 6 mice per group were treated via intranasal instillation with siRNA 18, siRNA 30, siRNA-Scramble (20 ug siRNA per mouse), or vehicle (saline) as the negative control. Intranasal delivery of naked siRNAs was performed 1 day before and 1, 3, and 5 days after SARS-CoV-2 Delta infection, with a day 6 endpoint. Weight and clinical scores were monitored daily. (**B**) Body weight of mice in each group. The body weight of each mouse was weighed and recorded daily. (**C**) Distribution of clinical score. The clinical score of each mouse was recorded on the 6th day of COVID-19 infection. Category 1: Low-grade clinical score; Category 2: Middle-grade clinical score; Category 3: High-grade clinical score. Statistical comparisons were performed using One-Way ANOVA. ** = *p* < 0.01, *** = *p* < 0.001, **** = *p* < 0.0001.

Previous studies have shown that functional naked siRNA can be successfully delivered to pulmonary cells by inhalation without the protection of a nanocarrier [18,19]. We therefore investigated the effect of naked siRNA intranasal administration using a combination of pre- and post-challenge with SARS-CoV-2 Delta variant in a K18-ACE2 transgenic mouse model of infection. Five groups (*n* = 6 mice per group) were included, with mice treated with siRNA 18, siRNA 30, or scrambled siRNA (20 µg per mouse) via intranasal instillation. Control groups included a SARS-CoV-2 vehicle control and a sham-treated, uninfected negative control (Figure 3A). Significantly less weight loss was reported in siRNA 18-treated mice with 8.07% (*p* < 0.01), compared to a 20.4% and 20.1% reduction in the SARS-CoV-2 siRNA Scram Ctrl group and SARS-CoV-2 no treatment group, respectively (Figure 3B). Additionally, both siRNA 18 and 30-treated mouse groups demonstrated improvement of clinical scores compared to control groups, with 100% (6/6 mice) and 83.33% (5/6 mice) in Category 1 with low clinical score, respectively, and only 50% (3/3 mice) and 66.66% (4/6 mice) in Category 1 in the siRNA control and the Sham non-virus treatment groups, respectively (Figure 3C).

**Figure 4:**
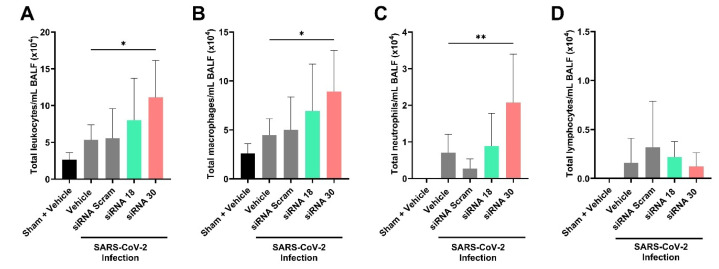
Effect of inflammatory cell infiltration in bronchoalveolar lavage fluid by naked siRNA treatment in vivo. The total amount of (**A**) leukocytes, (**B**) macrophages, (**C**) neutrophils, and (**D**) lymphocytes in the bronchoalveolar lavage fluid (BALF) of mice in each group was measured by cell counting six days after SARS-CoV-2 infection. Statistical comparisons were performed using One-Way ANOVA. * = *p* < 0.05, ** = *p* < 0.01.

Bronchoalveolar lavage fluid (BALF) was examined for the infiltration of immune cells and showed that siRNA 30 treatment resulted in significantly increased total leukocytes (11.1 × 10^4^/mL, *p* < 0.05), macrophages (8.93 × 10^4^/mL, *p* < 0.05), and neutrophils (2.08 × 10^4^/mL, *p* < 0.01) compared to siRNA 18 and control treatment groups (Figure 3C and Figure 4A). No increases in total lymphocytes were reported in any mouse groups (Figure 4D).

**Figure 5:**
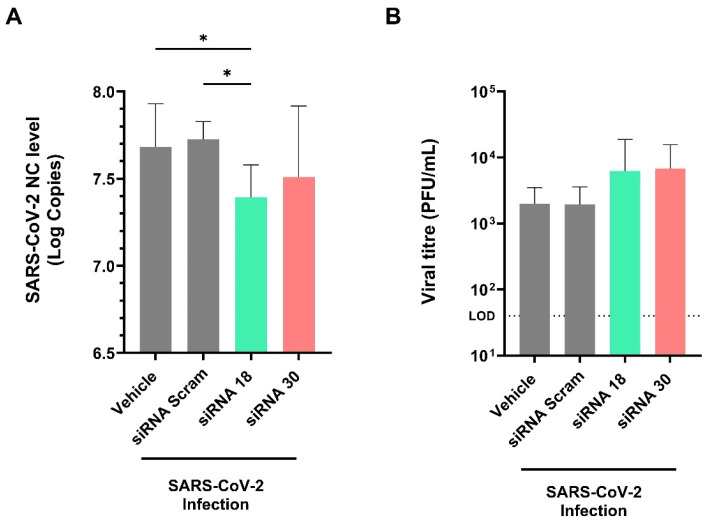
Effects of naked siRNAs on lung viral RNA and titres in SARS-CoV-2–infected K18-hACE2 mice. (**A**) The NC mRNA viral load in lung tissue of each mouse group was measured on day 6 post-infection. Lung tissue from mice was obtained for RNA extraction, and Luna^®^ Universal Probe One-Step RT-qPCR Kit (E3006) was used to detect the viral RNA levels. The results of each group were then analyzed, and statistical comparison was then performed as Log10 values. (**B**) Plaque assay for lung viral titre. After incubation with an overlay medium, plaques were visualized by crystal violet staining and quantified to determine viral titres, expressed as plaque-forming units (PFU) per lung. Statistical comparisons were performed using One-Way ANOVA. * = *p* < 0.05.

Importantly, siRNA 18 treatment led to a decrease in viral NC RNA levels in the lungs of infected mice by approximately half a log_10_, from 7.7 to 7.3 log_10_ copies (*p* < 0.05) compared to SARS-CoV-2 vehicle control (Figure 5A). While siRNA 18 significantly reduced viral RNA levels in lung tissue, no corresponding reduction in infectious viral titres was observed by plaque assay (Figure 5B), potentially due to differences in detection sensitivity. Despite siRNA 30 treatment improving clinical scores, there was no significant decrease in viral RNA levels or viral titres in the lung. Additionally, the timing of sample collection (day 6 post-infection) may coincide with a phase when viral replication is already declining, limiting detectable differences by plaque assay despite transcriptional suppression observed at the RNA level. This data demonstrate that intranasal delivery of naked siRNA can provide protection against SARS-CoV-2 infection in the K18-hACE2 mouse model.

### 2.4. Gene Silencing Effect of Chemically Modified siRNAs

**Figure 6:**
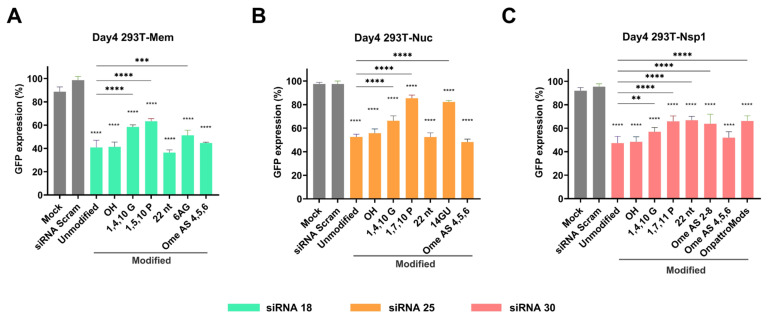
Assessment of siRNA chemical modifications for gene silencing. (**A**) Day 4 post-transfection of modified siRNA 18 in 293T Mem-GFP reporter cell lines showed maximal gene silencing. (**B**) Day 4 post-transfection of modified siRNA 25 in 293T Nuc-GFP reporter cell lines showed maximal gene silencing. (**C**) Day 4 post-transfection of modified siRNA 30 in 293T Nsp1-GFP reporter cell lines showed maximal gene silencing. All results were normalized with siRNA-Scramble with the corresponding cells. All experiments were performed in triplicate. Statistical comparisons were performed using One-Way ANOVA. ** = *p* < 0.01, *** = *p* < 0.001, **** = *p* < 0.0001.

As siRNA 30 treatment increased the number of infiltrating cells in the lung, we next explored whether chemical modifications improve the antiviral effect of our lead candidate siRNAs. The verified 293T lentiviral SARS-CoV-2 Nucleocapsid (Nuc), Membrane (Mem), and ORF1a-non-structural protein 1 (NSP1) reporter cell lines (Figure S1) were transfected using Lipofectamine RNAiMAX (Thermo Fisher Scientific, Waltham, MA, USA) with six different modifications on siRNA 18, six different modifications on siRNA 25, and seven different modifications on siRNA 30, respectively (Table 1). The modifications identified from these experiments were subsequently selected to further evaluate their capacity to minimize off-target induction ISGs. Gene silencing of GFP expression was assessed from day 2 to day 10 post-transfection using flow cytometry analysis. Alexa-Fluor647 labeled Scrambled siRNA was used to quantitate the percentage of siRNA positive 293T reporter cells, which peaked between day 2 and 4 post-transfection (Figure S2). The gene silencing effect on GFP expression was maximal at day 4 post-transfection (Figure S2). The chemical modifications of Overhang (OH, representing an siRNA with complementary 2nt 3′ overhangs on antisense strand), 22 nucleotides (22 nt, representing an siRNA with 3′ extended to 22 nt on both sense and antisense strands), and Ome-AS456 (2′O-methyl in position 4,5,6 of the antisense strand) on siRNA 18 and siRNA 25 suppressed GFP to a similar degree as unmodified siRNAs (Figure 6A,B), while Ome-AS456-modified and OH-modified siRNA 30 showed similar levels of GFP suppression compared with unmodified siRNA (Figure 6C). Interestingly, the maximal gene silencing effect of Ome-AS456-modified siRNA 30 and siRNA 18 was on both day 4 and day 5 post-transfection, which was extended by one day compared to unmodified siRNAs (Figure S2E). These results suggest that the three chemical modifications—OH, 22 nt, and Ome-AS456—enable siRNAs to perform similar gene silencing effects as unmodified siRNAs, and the Ome-AS456 modification has the potential to prolong the gene silencing effect of siRNAs. These three modifications were further examined for potential off-target effects via the type 1 interferon pathway induction of interferon-stimulated gene (ISG) effects and their antiviral effect against SARS-CoV-2 infection.

### 2.5. Chemical Modifications Reduce ISG Activation While Preserving Antiviral Efficacy of siRNAs

**Figure 7:**
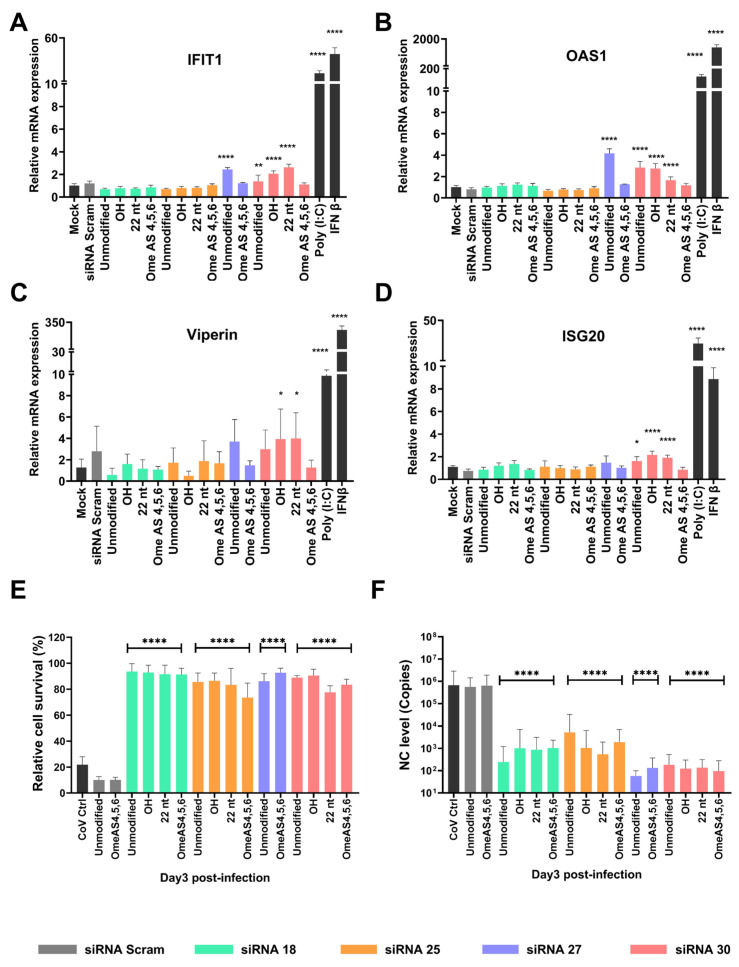
Assessment of siRNA chemical modifications for off-target ISG responses and antiviral effects. (**A**–**D**) Off-target ISG effects of siRNAs with or without modifications. HeLa T4+ cells were seeded 4 × 10^4^/well in 48-well plates and modified or unmodified siRNA 18, siRNA 25, siRNA 27, and siRNA 30 (5 nM) were transfected 6 h after cell seeding. Cells were lysed 72 h post-treatment and collected for RNA extraction. RT-qPCR was performed for (**A**) IFIT1, (**B**) OAS1, (**C**) Viperin, and (**D**) ISG20, and GAPDH was used as the housekeeping gene. Relative quantification was determined (2^−ΔΔCt^) and statistical comparisons were performed with mock, excluding both positive Ctrls, IFN β, and Poly (I:C), using One-Way ANOVA. (**E**,**F**) Antiviral effect of modified siRNAs against SARS-CoV-2 infection. Vero E6 cells were seeded 1.5 × 10^4^/well in 96-well plates, and modified or unmodified siRNA 18, siRNA 25, siRNA 27, and siRNA 30 (5 nM) were transfected 6 h after cell seeding. SARS-CoV-2 XBB.1.5 infection was then performed 1 h post-transfection. NucBlue was added to stain live cells and cells were imaged for the cell survival assay using the IN Cell Analyzer 72 h post-infection. (**F**) SARS-CoV-2 NC mRNA levels in cell culture supernatants measured by RT-qPCR. Statistical comparisons were performed with the infected siRNA-Scramble group using One-Way ANOVA. * = *p* < 0.05, ** = *p* < 0.01, **** = *p* < 0.0001.

To determine whether the chemical modification of OH, 22 nt, and Ome-AS456 has the potential to reduce ISG expression, we measured the mRNA levels of four ISGs in HeLa T4+ cells. No significant induction of ISGs was observed with siRNA 18 and siRNA 25, regardless of modification status (Figure 7A–D). However, siRNA 27 treatment led to increased expression of IFIT1 (Figure 7A) and OAS1 (Figure 7B), and siRNA 30 induced elevated expression of IFIT1 (Figure 7A), OAS1 (Figure 7B), and ISG20 (Figure 7D). Interestingly, when siRNA 30 was modified with either 2′-OH or 22-nt truncation, all four ISGs—including Viperin, which was not elevated by the unmodified siRNA 30—showed increased mRNA levels (Figure 7C). Notably, the Ome-AS456 modification of siRNA 27 and 30 significantly decreased the ISG expression to levels comparable to controls.

The three chemical modifications were further explored using the cell survival assay to determine whether siRNAs with the three selected modifications continue to provide protection against SARS-CoV-2 XBB.1.5 infection in Vero E6 cells, which demonstrated similar levels of cell survival for both modified and non-modified siRNA (Figure 7E). To further evaluate whether antiviral activity was preserved following chemical modification, the Ome-AS456 versions of the four lead siRNAs were examined under the same experimental conditions. At a fixed dose of 5 nM, Ome-AS456–modified siRNAs showed improved cell survival at day 3 post-infection compared with their unmodified counterparts (Figure 7E). Consistent with the cell survival results, Ome-AS456-modified siRNAs also produced significant suppression of viral RNA as unmodified siRNAs (Figure 7F), indicating that the reduced immune activation associated with this modification does not compromise antiviral function.

**Figure 8:**
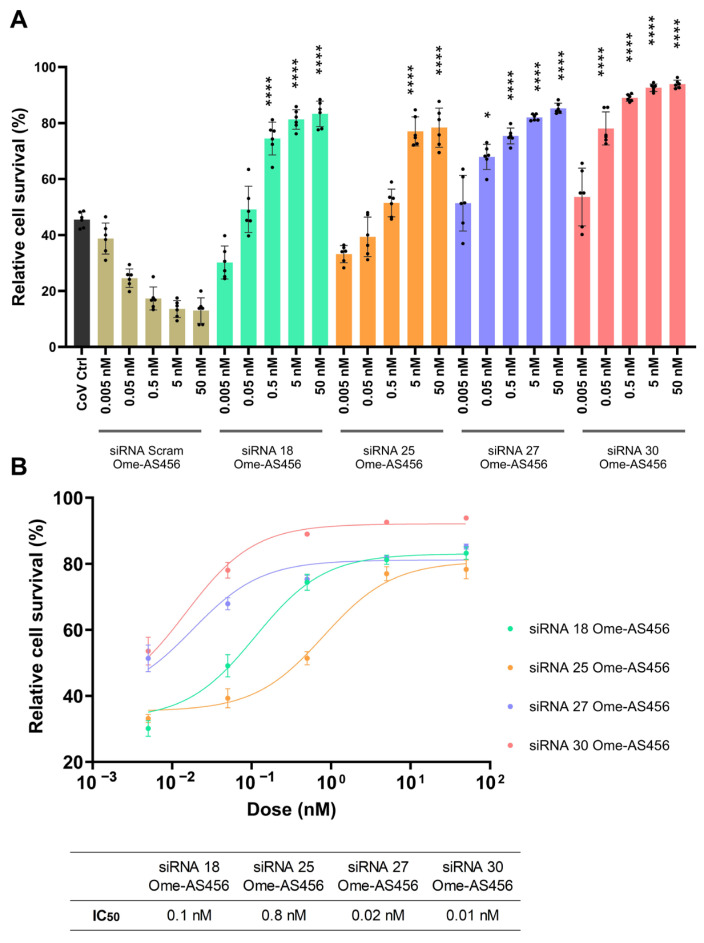
Dose-response analysis of 2′-O-methyl–modified siRNAs (siRNA Ome-AS456). Vero E6 cells were treated with serial dilutions of siRNA 18, 25, 27, or 30 (0.005–50 nM) and infected with SARS-CoV-2 XBB.1.5. (**A**) Relative cell survival in SARS-CoV-2–infected Vero E6 cells treated with siRNA Ome-AS456 (18, 25, 27, or 30) across increasing concentrations (0.005 nM to 50 nM). Statistical comparisons were performed with siRNA Scramble (Scram) control group with the same siRNA concentration. * = *p* < 0.05, **** = *p* < 0.0001. (**B**) Dose-response curves of siRNA Ome-AS456 against SARS-CoV-2 XBB.1.5 infection. The IC_50_ values were calculated from nonlinear regression curves, reflecting the concentration at which each siRNA achieved 50% of its maximal protective effect. Data represent mean ± SD from six independent experiments.

To more comprehensively assess the antiviral potency of the modified siRNAs, a full dose-response assay was performed across a concentration range of 0.005–50 nM. All Ome-AS456–modified siRNAs produced clear dose-dependent improvements in cell survival upon SARS-CoV-2 infection (Figure 8A). Nonlinear regression analysis revealed low IC_50_ values, 0.1 nM (siRNA 18), 0.8 nM (siRNA 25), 0.02 nM (siRNA 27), and 0.01 nM (siRNA 30), demonstrating that the Ome-AS456 modification preserves or enhances antiviral potency relative to the unmodified forms (Figure 8B). Together, these findings confirm that site-specific 2′-O-methyl modification minimizes innate immune activation while maintaining strong antiviral activity in vitro.

## 3. Discussion

Since the emergence of SARS-CoV-2, various antiviral therapeutics have been developed and approved [28,29,30,31,32,33,34,35], yet their efficacy has been increasingly challenged by the rapid evolution of viral variants [36,37,38,39,40,41,42]. To address this, four lead candidate anti-COVID siRNAs were developed, each targeting highly conserved, distinct genomic regions of SARS-CoV-2 that exhibit broad-spectrum activity against circulating and emerging variants [17]. These siRNA sequences have been reported in our previous work, and in the present study, in vivo effects were explored as a proof-of-concept foundation, together with site-specific chemical modifications, to alleviate the innate immune activation typically associated with unmodified siRNAs.

To evaluate the antiviral potency of these lead siRNAs, dose-response analyses were conducted in SARS-CoV-2-infected cells. While all four siRNAs (18, 25, 27, and 30) were designed with complete sequence complementarity to conserved regions of the viral genome (Figure 1), their antiviral effects varied substantially across different SARS-CoV-2 strains. For instance, in XBB.1.5-infected Vero E6 cells, siRNA 27 and siRNA 30 achieved potent virus inhibition at a minimum effective dose of 0.05 nM, whereas siRNA 18 required 0.5 nM and siRNA 25 required 5 nM to achieve comparable effects (Figure 2). These findings demonstrate that sequence complementarity alone is insufficient to ensure consistent RNAi efficacy, highlighting the importance of functional screening in multiple viral and cellular contexts, as supported by previous studies [17,43]. It should also be noted that the apparent discrepancy between cell survival and viral RNA reduction in vitro may, at least in part, reflect the intrinsic cytotoxicity of Lipofectamine, which reduces baseline cell viability and thereby underestimates the protective effect of siRNAs in survival-based readouts. This observation further highlights the importance of developing and employing biocompatible delivery systems for future studies to minimize delivery-associated toxicity and more accurately evaluate the potential antiviral activity of siRNAs.

To determine the in vivo antiviral efficacy, two lead candidate siRNAs were assessed in a K18-hACE2 transgenic mouse model of SARS-CoV-2 Delta infection. SiRNA 18 induced attenuation of disease progression when administered via intranasal instillation, as shown by reduced clinical scores and less weight loss compared to the untreated infected control group (Figure 3). Furthermore, siRNA 18 treatment led to a modest reduction of approximately 0.5 log_10_ in viral RNA levels in lung tissues by day 6 post-infection (Figure 5). However, this protective effect remained modest in comparison to uninfected controls, underscoring the limited stability and in vivo bioavailability of unmodified siRNAs when delivered without a protective carrier [44,45].

Interestingly, siRNA 30, which showed strong in vitro antiviral effects, was associated with a marked increase in BALF leukocyte infiltration in vivo, particularly macrophages and neutrophils (Figure 4). This in vivo immune cell recruitment may reflect the off-target immune activation potential of siRNA 30 observed in vitro. Specifically, siRNA 30 induced elevated expression of multiple ISGs, including IFIT1, OAS1, and ISG20 (Figure 6). This suggests that siRNA 30 may contribute not only to antiviral activity but also to undesirable proinflammatory responses in vivo. This immunostimulatory profile highlights the dual role of siRNAs as both antivirals and modulators of host innate immune responses, underscoring the importance of carefully balancing efficacy with immunological safety in therapeutics.

It is worth noting the significant difference observed in lung viral RNA (as measured by RT-qPCR) and the lack of a decrease in infectious viral titres in BALF (as measured by plaque assay) (Figure 5). One explanation is the saturation of siRNA in the lung may be too low to inhibit viable virus at a detectable level by plaque assay, while RT-qPCR is more sensitive. This may reflect a threshold effect, where siRNA concentrations, although capable of reducing viral RNA in mice lungs, may be inadequate to fully suppress the replication-competent viral population in the BALF, or to reach the threshold required for a measurable reduction in infectious virus detectable by PFU assay in the airways. The differing assay kinetics may also contribute to this discrepancy, wherein lower viral loads in some mice may have increased to match the initially higher loads in others over the span of the replication window at day 6 post-infection, thereby reducing the measurable differences in infectious titres by the time of plaque assay. Additionally, RT-qPCR is inherently more sensitive and may capture early or partial effects of gene silencing [46,47], whereas plaque assays reflect only viable virus capable of productive infection. While RT-qPCR offers a sensitive and valuable tool for virological analysis, it cannot replace plaque assays in assessing infectious viral burden in practical applications [48]. Additional factors such as insufficient siRNA saturation in the lung and the absence of natural history profiling of viral kinetics may also have contributed.

As this study represents a proof-of-concept investigation, detailed biodistribution, pharmacokinetic, and histopathology analyses were not performed, and future studies using stabilized, carrier-based formulations will incorporate these assessments to better characterize in vivo kinetics and immunopathology. Although these antiviral effects remain preliminary, they nevertheless demonstrate that unmodified siRNAs can exert measurable in vivo activity, and importantly, reveal their immunological consequences, particularly the proinflammatory response induced by siRNA30. The primary contribution of this work lies in demonstrating the immunological consequences of unmodified siRNAs and highlighting the potential benefit of chemical modification as a strategy to mitigate these effects. These observations collectively reinforce the need to further improve siRNA delivery efficiency—potentially through incorporation of carriers. Such strategies are particularly relevant for immunocompromised or immunosuppressed patients, where conventional antivirals or vaccines may have limited efficacy or safety, positioning siRNA therapeutics as a potentially valuable complementary approach.

To minimize potential immune activation, various chemical modifications of the siRNAs were also investigated, with particular focus on the 2′-Ome-AS456 modification, which incorporates 2′-O-methyl substitutions at positions 4, 5, and 6 of the antisense strands [26]. This 2′-Ome-AS456 modification effectively minimizes ISG-related off-target responses while preserving the antiviral potency of siRNAs against SARS-CoV-2 (Figure 7). Moreover, when compared with the IC_50_ values of the unmodified siRNAs (Figure 2D), the Ome-AS456 variants showed similar or further improved potency across all four siRNA candidates (Figure 8B), further supporting that this modification preserves or enhances silencing efficiency while reducing immune-stimulation. These findings are consistent with previous studies demonstrating that 2′-O-methyl modifications at the 3′-end and the central region of the antisense strand can enhance siRNA activity [49,50]. However, limited data have been available regarding their influence on ISGs activation [51,52]. Interestingly, 2′-O-methyl modification of 3-mer RNA fragments, such as GAC and GAG, are known to be potent TRL7 and TRL8 antagonists, respectively, that reduce TRL7 sensing in vivo [53]. Both siRNA 27 and 30 have the same 3-mer GAC sequence at position 4, 5, and 6 of the antisense strands, suggesting that this TRL7 antagonist effect may have contributed to the observed reduction of ISG expression. This potential effect warrants further investigation and may provide additional benefits for utilizing 2′-O-methyl chemical modification in antiviral siRNA therapeutic design. Additionally, various chemical modifications, such as 2′-fluoro, phosphorothioate, and 2′-O-methyl, have been widely applied to enhance siRNA stability and reduce innate immune activation [18,54]. A recent study by Khvorova et al. using fully chemically stabilized, multimeric siRNA architectures has demonstrated that optimized scaffolds can achieve robust lung delivery and pronounced antiviral efficacy in mouse models of SARS-CoV-2 infection [55]. In contrast, the present study focuses on naked and minimally modified siRNAs as a mechanistic, proof-of-concept investigation, delineating sequence- and modification-dependent effects on antiviral activity and innate immune stimulation that are complementary to such stabilized delivery platforms. Phosphorothioate and 2′-O-methyl modifications have been shown to significantly improve siRNA stability in vitro [56]. In particular, the position-specific modifications have been emphasized as a key determinant of both silencing efficacy and off-target immune activation [57]. Similarly, our study supports that site-specific 2′-O-methyl modifications can be utilized as an effective approach to attenuate immunostimulatory off-target effects while preserving antiviral potency. Interestingly, scrambled siRNA controls exhibited reduced cell survival at higher doses, which may reflect sequence-independent cytotoxicity associated with Lipofectamine-mediated delivery [58]. Such effects have also been reported previously for non-targeting siRNAs and likely reflect dsRNA-triggered stress responses or delivery-related toxicity rather than antiviral activity [59].

A limitation of the present study is that the in vivo efficacy and immune-modulatory benefits of the chemically modified siRNAs were not evaluated; therefore, these results should be interpreted within a proof-of-concept framework. As the in vivo evaluation of the 2′-O-methyl–modified siRNAs was not performed in this work, future studies will assess the Ome-AS456 variants in vivo using stabilized, carrier-based formulations to determine their therapeutic potential. Nevertheless, our findings establish an important mechanistic basis for future investigations, highlighting the need for in vivo validation of chemically modified siRNAs as a critical next step toward therapeutic translation.

Taken together, these results describe the development of a highly specific siRNA-based antiviral strategy against SARS-CoV-2 and provide proof-of-concept evidence supporting its activity following intranasal delivery. Beyond demonstrating antiviral activity, this study also provides insight into the immunomodulatory consequences of siRNA therapy. Optimization via chemical modifications, particularly 2′-Ome-AS456, is crucial for minimizing off-target immune activation, highlighting the critical role of site-specific chemical modification in balancing siRNA efficacy and off-target responses. The highly conserved sequences that confer resilience against viral mutations, the potent antiviral efficacy observed at low IC_50_ values, and the capacity to reduce innate immune activation through appropriate chemical modifications collectively support the future potential of these siRNAs into the broader spectrum of therapeutics in the post-pandemic era.

## 4. Materials and Methods

### 4.1. Animals

A total of 30 K18-hACE2 transgenic mice were used in this study (bred in-house at Centenary Institute, Sydney). All animal ethics procedures were approved under the approval number AEC 2020-019, with biosafety approval provided by the Sydney Local Health District (IBC 20-051). Mice were provided ad libitum access to a standard chow diet and water and were maintained on a 12 h light–dark cycle.

### 4.2. Cell Culture

HEK293T cells were cultured in Dulbecco’s modified Eagle’s medium (DMEM, Gibco™, Thermo Fisher Scientific, Waltham, MA, USA Cat#11995065) with 10% heat-inactivated fatal bovine serum (FBS) and 100 U/mL Penicillin-Streptomycin (Pen-Strep, Gibco™, Thermo Fisher Scientific, Waltham, MA, USA Cat#15140122). HeLa T4+ cells were cultured in DMEM with 10% heat-inactivated FBS, 100 U/mL Pen-Strep, and 500 µg/mL G418 (Gibco™, Thermo Fisher Scientific, Waltham, MA, USA Cat#10131027). Vero E6 cells were cultured in Eagle’s Minimum Essential Medium (EMEM, Gibco™, Thermo Fisher Scientific, Waltham, MA, USA, Cat#11095080) with 10% heat-inactivated FBS, 1 mM sodium pyruvate (Gibco™, Thermo Fisher Scientific, Waltham, MA, USA Cat#11360070), and 100 U/mL Pen-Strep.

### 4.3. Viruses

The virus isolates used in both in vitro and in vivo siRNA screening include hCoV-19/Australia/NSW4605/2021 (EPI_ISL_1911187) Delta and hCoV-19/Australia/NSW-ICPMR-44477/2023 (EPI_ISL_17408981) Omicron XBB.1.5.

### 4.4. Conservation Analysis

A total of 3543 SARS-CoV-2 and SARS-CoV-1 sequences (3010 SARS-CoV-2 human sequences, 232 SARS-CoV-2 animal sequences, 270 SARS-CoV-1 human sequences, and 31 SARS-CoV-1 animal sequences) were obtained from both NCBI and GISAID. MAFFT alignments were performed using Geneious Prime (version 2023.2.1), and a heat-map was generated from blue (percentile < 10) to yellow (percentile 10–90) and red (percentile > 90) using the average identity of each 19 bp sequence.

Conservation analysis was performed to evaluate the sequence stability of our four lead siRNA candidates across multiple SARS-related coronavirus genomes from both human and animal hosts. The analysis assessed the proportion of sequences that completely matched each siRNA across diverse viral strains to determine their potential to remain effective against existing and evolving variants.

### 4.5. Construction and Verification of SARS-CoV-2 Lentiviral-GFP Reporter Cell Lines

Synthetic SARS-CoV-2 DNA fragments of 5′UTR, ORF1a-Nsp1, Membrane, Nucleocapsid, Envelope, or Spike were synthesized (Genewiz, Azenta Life Sciences, South Plainfield, NJ, USA), and lentiviral constructs expressing individual fragments driving GFP for the 5′UTR or fused to GFP for the remaining fragments were generated via conventional cloning strategies.

Lentivirus was generated using a second-generation lentiviral system. In brief, the lentiviral vector DNA, psPAX2 packaging, and VSV-G envelope plasmid DNA were combined at a ratio of 4:4:1. After 72 h of culturing, Virus-containing media (VCM) was harvested, and viral stock titres were then analyzed. 293T cells were then infected with lentiviral-SARS-CoV-2-GFP, then Blasticidin selection (20 µg/mL) and cell sorting were performed to acquire Lenti-SARS-CoV-2-GFP reporter cell lines with the relatively higher expression rates of GFP.

To verify Lenti-SARS-CoV-2-GFP reporter cell lines, candidate siRNA- 18, 25, 27, 30 at a concentration of 5 nM were transfected into their respective 293T-GFP reporter cell line using Lipofectamine RNAiMAX (Invitrogen™, Thermo Fisher Scientific, Waltham, MA, USA; Cat#13778100). GFP expression was then analyzed using flow cytometry to identify the downregulation effect of candidate siRNAs on their targeted genes of reporter cells (Figure S1).

### 4.6. siRNA Transfection Using Lipofectamine

All siRNA were synthesized at the UNSW RNA Institute. Transfection of siRNA (5 nM or as indicated) was performed using Lipofectamine RNAiMax (Invitrogen™, Thermo Fisher Scientific, Waltham, MA, USA; Cat#13778100) according to the manufacturer’s protocol. Cell fixation and flow cytometry were then performed on designated days.

### 4.7. Flow Cytometry and Cell Sorting

After resuspending the cells and centrifuging (300× *g*, 5 min), cells were fixed in 2% of paraformaldehyde for 30 min at room temperature. Flow cytometry was then performed using the BD LSRFortessa SORP (BD Biosciences, San Jose, CA, USA) with at least 20,000 cells per sample. For cell sorting, cells were resuspended and centrifuged (300× *g*, 5 min), then resuspended again in Flow Cell Sorting buffer (PBS, EDTA 2 mM, heat-inactivated FBS 10%) before filtration using 5 mL falcon tubes with cell strainer snap cap. After cell sorting, sorted cells were collected using 2–5 mL cell media with 20% heat-inactivated FBS.

### 4.8. Protective Effect of siRNAs Against SARS-CoV-2

Vero E6 cells were seeded into 96-well-plates and then transfected with siRNA 5 nM delivered by Lipofectamine 2 h pre-SARS-CoV-2 infection. Supernatant was collected after cell survival assay. RNA extraction (Monarch Total RNA Miniprep Kit, New England Biolabs, Ipswich, MA, USA; Cat# T2010S) and RT-qPCR (Luna^®^ Universal Probe One-Step RT-qPCR Kit, New England Biolabs, Ipswich, MA, USA; E3006) were then performed to measure the virus nucleocapsid (NC) mRNA levels according to the manufacturer’s protocol.

### 4.9. Dose-Response Assay for siRNAs and LNP-siRNAs

Vero E6 cells were seeded into 96-well plates before being transfected with siRNA at 0.005, 0.05, 0.5, 5, and 50 nM by Lipofectamine or 25, 50, and 100 nM when delivered by LNPs. SARS-CoV-2 infection was then performed on the same day as siRNA transfection. NucBlue was added 3 days post-treatment, and cell survival was analyzed by the IN Cell Analyzer 2500 HS (Cytiva, Marlborough, MA, USA) plate reader and IN Carta Image Analysis Software (version 1.15, Cytiva, Marlborough, MA, USA).

### 4.10. Cell Survival Assay

Cells were stained using NucBlue (Invitrogen™, Thermo Fisher Scientific, Waltham, MA, USA; Cat#R37605) according to manufacturer’s protocol 24 h prior to imaging. Then images were acquired for brightfield (white light) and blue light spectrums by an IN Cell Analyzer 2500 HS plate reader with a 10× zoom lens. Cell survival was analyzed by IN Carta Image Analysis Software to determine nuclei counts of live cells for the quantification.

### 4.11. In Vivo Assessment of siRNA Treatment in a SARS-CoV-2 Mouse Model of Infection

Female K18-hACE2 transgenic mice (Centenary Institute, Sydney, Australia) were divided into five groups (*n* = 6 per group) to evaluate the protective efficacy of naked siRNAs against SARS-CoV-2 infection. Briefly, mice were anesthetized with isoflurane and intranasally administered siRNA-18, siRNA-30, siRNA-Scramble (20 μg siRNA per mouse), or received PBS as the negative control. Intranasal treatments were administered on day –1 (1 day pre-infection) and on days 1, 3, and 5 post-infection. Mice were housed in the Centenary Institute PC3 facility under specific pathogen-free conditions and monitored daily for body weight and clinical scores. On day 6 post-infection, mice were euthanized with sodium pentobarbitone and endpoint procedures were completed as previously described [60,61,62]. Blood was collected via heart bleed, multi-lobe lungs were tied off, and the single left lobe was lavaged with 1 mL of HANKS solution (Sigma-Aldrich, St. Louis, MO, USA). Bronchoalveolar lavage fluid (BALF) was collected to measure total leukocytes, with differential counts performed to enumerate macrophages, neutrophils, and lymphocytes. Multi-lobe lungs were collected, and lung tissues were homogenized in HANKS solution to liberate the virus from tissue for plaque assay viral titre enumeration. The lung tissue cell pellet was resuspended in TRIZOL for RNA extraction. Viral RNA levels were quantified by RT-qPCR targeting the nucleocapsid (NC) gene using the Luna^®^ Universal Probe One-Step RT-qPCR Kit (New England Biolabs, Ipswich, MA, USA; Cat#E3006).

### 4.12. Off-Target Effect of siRNAs

To evaluate potential off-target immune activation, we assessed the expression of four interferon-stimulated genes (ISGs) commonly used as markers of type I interferon responses triggered by double-stranded RNA, as previously reported [63]. HeLa T4+ cells were seeded in 48-well plates and transfected with candidate siRNAs 5 nM for 72 h. Transfection of HeLa T4+ cells occurred 6 h after seeding. Positive controls included IFN *β* added 24 h before cell collection at 500 IU/mL, and Poly(I:C) transfected at 0.1 µg/mL 6 h before cell collection using Lipofectamine RNAiMAX according to the manufacturer’s protocol. After cell collection, RNA extraction was performed using a Monarch Total RNA Miniprep Kit (New England Biolabs, Ipswich, MA, USA, Cat#T2010S), and mRNA expression levels of four type I IFN response genes—ISG20, Viperin, IFIT1, and OAS1—were measured by SYBR-Green–based quantitative real-time PCR assays using the Luna^®^ Universal One-Step RT-qPCR Kit (New England Biolabs, Ipswich, MA, USA; Cat#E3005), with GAPDH as the housekeeping gene.

### 4.13. siRNA Modifications

Various modified COVID siRNAs, including overhang (OH) [20], mismatches [21,22], 22 nt-extended (22 nt) [21], and 2′O-methylation (Ome), were designed [18,23,24,25,26] (Table 1). Based on our previous conservation analysis, the virus sequence targeted by siRNA18 mainly underwent 6A > G mutations between August and October 2022, while the virus sequence targeted by siRNA25 mainly reported 14G > U mutations between November and December 2022 [17]. Thus, we also designed modified siRNAs targeting these two mutations (CoV-18-6AG and CoV-25-14GU), which corrected the bases at the mutation sites. Details of each modified siRNA are shown in Table 1.

### 4.14. Statistical Analysis

Alignments of sequences were performed using the Geneious Prime software (version 2023.2.1). Data were analyzed as mean ± SEM from at least three independent experiments using one-way analysis of variance (ANOVA). For mouse experiments, body weights were analyzed using a two-way ANOVA, while leukocyte counts were analyzed using a One-Way ANOVA. Statistical analysis was performed with GraphPad Prism software (version 10.2.3). * *p* < 0.05, ** *p* < 0.01, *** *p* < 0.001, **** *p* < 0.0001.

## 5. Conclusions

This study provides proof-of-concept evidence for the antiviral activity of our anti-COVID siRNAs for the treatment of SARS-CoV-2 infection, while also emphasizing the importance of balancing antiviral efficacy with host immunological responses through the use of conserved siRNAs and chemical modifications. The modest in vivo antiviral efficacy via intranasal administration of siRNAs supports the feasibility of direct respiratory delivery and provides insight into how siRNAs may not only suppress viral replication but also modulate innate immune activation. These findings establish an initial foundation to the development of siRNA therapeutics employing nanocarrier–based delivery systems to further improve the stability, bioavailability, and future translational potential of conserved, chemically modified siRNA candidates in the context of viral immunomodulation.

## 6. Patents

A.D.K., S.L., Y.Z., and C.L.A. hold patents for antiviral siRNAs targeting SARS-CoV-2.

## Figures and Tables

**Table 1:** Sequences of modified top-four siRNAs shown to protect against SARS-CoV-2.

Modified CoV siRNAs	Strand	Sequence (5′–3′)	Target Genes	Ref.
SiRNA-18 -OH	Sense Antisense	GCUACAUCACGAACGCUUU [dT][dT] AAAGCGUUCGUGAUGUAGCAA	Membrane	[20]
SiRNA-18 -1,4,10 G	Sense Antisense	GCUACAUCAGGAACGGUUA [dT][dT] AAAGCGUUCGUGAUGUAGC [dT][dT]	Membrane	[21]
SiRNA-18 -1,5,10 P	Sense Antisense	UCUAUAUCAUGAACGCUUU [dT][dT] AAAGCGUUCGUGAUGUAGC [dT][dT]	Membrane	[21,22]
SiRNA-18 -22 nt	Sense Antisense	GCUACAUCACGAACGCUUUCUU GAAAGCGUUCGUGAUGUAGCAA	Membrane	[21]
SiRNA-18 -6AG	Sense Antisense	GCUACGUCACGAACGCUUU [dT][dT] AAAGCGUUCGUGACGUAGC [dT][dT]	Membrane	[17]
SiRNA-18 -Ome AS 4,5,6	Sense Antisense	GCUACAUCACGAACGCUUU [dT][dT] AAAmGmCmGUUCGUGAUGUAGC [dT][dT]	Membrane	[18,23,24,25,26]
SiRNA-25 -OH	Sense Antisense	GGGUUGCAACUGAGGGAGC [dT][dT] GCUCCCUCAGUUGCAACCCAU	Nucleocapsid	[20]
SiRNA-25 -1,4,10 G	Sense Antisense	GGGUUGCAAGUGAGGCAGG [dT][dT] GCUCCCUCAGUUGCAACCC [dT][dT]	Nucleocapsid	[21]
SiRNA-25 -1,7,10 P	Sense Antisense	UGGUUGUAAUUGAGGGAGC [dT][dT] GCUCCCUCAGUUGCAACCC [dT][dT]	Nucleocapsid	[21,22]
SiRNA-25 -22 nt	Sense Antisense	GGGUUGCAACUGAGGGAGCCUU GGCUCCCUCAGUUGCAACCCAU	Nucleocapsid	[21]
SiRNA-25 -14GU	Sense Antisense	GGGUUGCAACUGATGGAGC [dT][dT] GCUCCAUCAGUUGCAACCC [dT][dT]	Nucleocapsid	[17]
SiRNA-25 -Ome AS 4,5,6	Sense Antisense	GGGUUGCAACUGAGGGAGC [dT][dT] GCUmCmCmCUCAGUUGCAACCC [dT][dT]	Nucleocapsid	[18,23,24,25,26]
SiRNA-27 -Ome AS 4,5,6	Sense Antisense	CGAGAAAACACACGUCCAA [dT][dT] UUGmGmAmCGUGUGUUUUCUCG [dT][dT]	ORF1ab (NSP1)	[18,23,24,25,26]
SiRNA-30 -OH	Sense Antisense	GGCAUUCAGUACGGUCGUA [dT][dT] UACGACCGUACUGAAUGCCUU	ORF1ab (NSP1)	[20]
SiRNA-30 -1,4,10 G	Sense Antisense	GGCAUUCAGAACGGUGGUC [dT][dT] UACGACCGUACUGAAUGCC [dT][dT]	ORF1ab (NSP1)	[21]
SiRNA-30 -1,7,11 P	Sense Antisense	UGCAUUUAGUAUGGUCGUA [dT][dT] UACGACCGUACUGAAUGCC [dT][dT]	ORF1ab (NSP1)	[21,22]
SiRNA-30 -22 nt	Sense Antisense	GGCAUUCAGUACGGUCGUAGUG CUACGACCGUACUGAAUGCCUU	ORF1ab (NSP1)	[21]
SiRNA-30 -Ome AS 2–8	Sense Antisense	GGCAUUCAGUACGGUCGUA [dT][dT] UmAmCmGmAmCmCmGUACUGAAUGCC [dT][dT]	ORF1ab (NSP1)	[18,23,24,25,26]
SiRNA-30 -Ome AS 4,5,6	Sense Antisense	GGCAUUCAGUACGGUCGUA [dT][dT] UACmGmAmCCGUACUGAAUGCC [dT][dT]	ORF1ab (NSP1)	[18,23,24,25,26]
SiRNA-30-OnpattroMods	Sense Antisense	G*mGC*A*mU*mUC*A*G*U*A*mCG*mGmUmCmGU*mA[dT][dT] U*A*C*G*A*C*mCG*U*A*C*U*G*A*A*U*mGC*C*[dT][dT]	ORF1ab (NSP1)	[27]

-OH, Complementary RNA 3′-Overhang of antisense strand. -22 nt, 3′ extended to 22 nt of both sense and antisense strand. -1,4,10 G/-1,5,10 P/-1,7,11 P, Mismatches in passenger strand (counted from guide strand positions). -6AG, A > G in the 6 position of target sequence; -14GU, G > U in the 14 position of target sequence. -Ome AS 2–8, 2′O-methylation in the 2–8 position of the antisense strand. -Ome AS 4,5,6, 2′O-methylation in the 4,5,6 position of the antisense strand. -OnpattroMods, Phosphonothioate linkages and 2′O-methyl of both the sense and antisense strands to match Onpattro formulation. mA/mG/mC/mU, 2′O-methyl RNA. [dT], DNA bases within RNA oligos. * Phosphorothioate linkages.

## Data Availability

The original contributions presented in this study are included in this article. Further inquiries can be directed to the corresponding author.

## Supplementary Materials

The following supporting information can be downloaded at https://www.mdpi.com/article/10.3390/ijms262311675/s1.

## Abbreviations

The following abbreviations are used in this manuscript:

SARS-CoV-2	Severe Acute Respiratory Syndrome Coronavirus 2
siRNA	Small interfering RNA
RNAi	RNA interference
BALF	Bronchoalveolar lavage fluid
ISG	Interferon-stimulated gene
