# Supplementary material for: Respiratory Delivery of Highly Conserved Antiviral siRNAs Suppress SARS-CoV-2 Infection
Source: Int J Mol Sci. 2025 Dec 2;26(23):11675. doi: 10.3390/ijms262311675 (PMC12692593; doi:10.3390/ijms262311675)
Supplement: Supplementary file 1 [file ijms-26-11675-s001.zip › ijms-3969758-supplementary.pdf]

International Journal of
Molecular Sciences

MDPI

Supplementary Materials

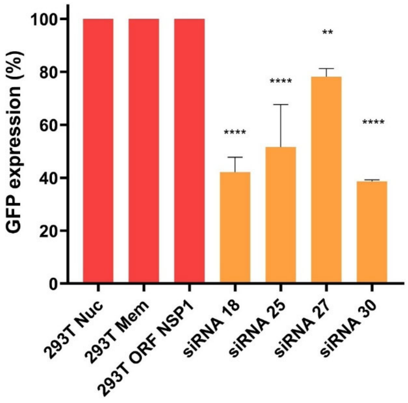

Figure S1. Verification of 293T lentiviral SARS-CoV-2 reporter cell lines. 293T reporter cell lines expressing SARS-CoV-2 Nucleocapsid, Membrane, and Non-structural protein 1(ORF1a-NSP1) were transfected with 5 nM siRNA18, siRNA 25, siRNA 27 and siRNA 30 (produced by UNSW RNA Institute) using Lipofectamine RNAiMAX (Invitrogen, USA). After 48 hours of transfection, cell fixation with 2% paraformaldehyde and flow cytometry was then performed for GFP expression. All results were normalized with GFP expression of targeted 293T-GFP-reporter cells. **p<0.005 ****p<0.0001.

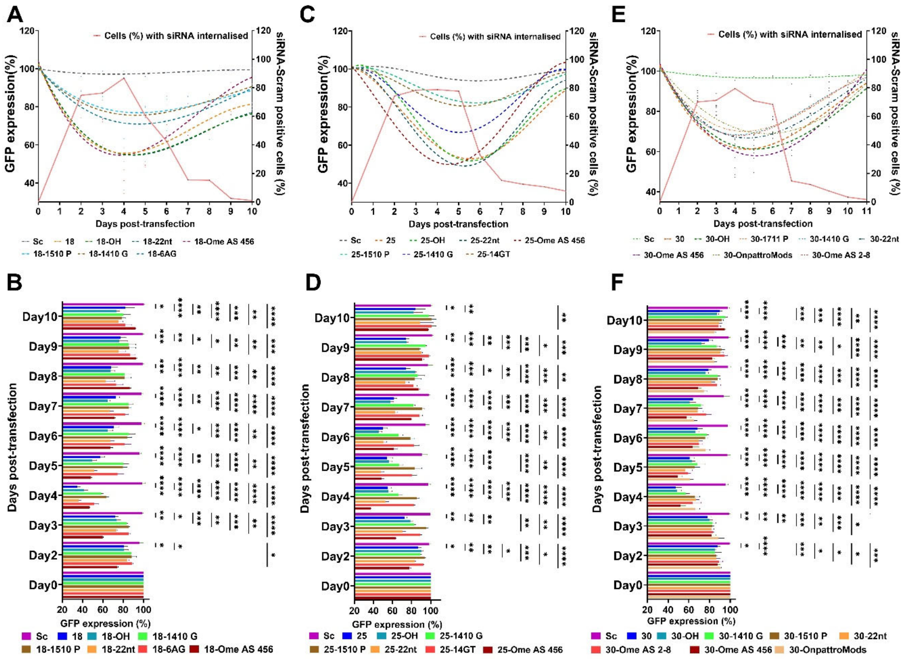

Figure S2. Down-regulation of GFP expression by modified COVID siRNAs in 293T Mem, Nuc, or Nsp1-GFP reporter cell lines from 2 days to 10 days post-transfection. (A, B) Down-regulation of GFP expression and AF647-labelled siRNA-Scramble positive cells of modified COVID siRNAs 18 (5 nM) in 293T Mem-GFP reporter cell lines. (C, D) Down-regulation of GFP expression and AF647-labelled siRNA-Scramble positive cells of modified COVID siRNAs 25 (5 nM) in 293T Nuc-GFP reporter cell lines.(E, F) Down-regulation of GFP expression and AF647-labelled siRNA-Scramble positive cells of modified COVID siRNAs 30 (5 nM) in 293T Nsp1-GFP reporter cell lines. All results were normalized with AF647-labelled siRNA-Scramble with the same concentration. All experiments were performed in triplicate. Statistical comparisons were performed using One-Way ANOVA. * = p < 0.05, ** = p < 0.01, *** = p < 0.001, **** = p < 0.0001.
